# EuroBioForum - the gateway to partnering in Europe

**DOI:** 10.1186/1878-5085-5-S1-A5

**Published:** 2014-02-11

**Authors:** Wouter Spek

**Affiliations:** 1 EuroBioForum, The Netherlands

## 

EuroBioForum brings people and parties together, initiating annual conferences and developing a strong community leading to networking and partnering. We aim to assure the position of Europe as the research and implementation capital of Personalised Medicine, all for the benefit of the patient.

## Our target groups for partnerships are

national governments, bioclusters, regional initiatives, funding organisations, national research councils, patient organisations, scientific associations, industrial federations and the European Commission.

## Personalised Medicine needs involvement of all stakeholders

How can we address the challenges to find solutions? The complexity of diseases and the biology of humans are calling for a different approach. But the question is whether the current focus of Personalised Medicine is the solution. What are current perspectives in policy making and funding of Personalised Medicine? How can the aforementioned issues be addressed? How to tackle the future challenges in Personalised Medicine?

EuroBioForum tries to provide insight into current initiatives and key players in Personalised Medicine in Europe. From this starting point, strategic alliances and partnerships will be facilitated to empower the field, to warrant scientific, economic and societal success and to capitalise on European added value. To do so, EuroBioForum aims to align the priorities of both funding and research programmes at national and regional level, in order to get the most out of every euro and out of everyone’s effort.

